# Structural and functional deficits and couplings in the cortico-striato-thalamo-cerebellar circuitry in social anxiety disorder

**DOI:** 10.1038/s41398-022-01791-7

**Published:** 2022-01-21

**Authors:** Xun Zhang, Xueling Suo, Xun Yang, Han Lai, Nanfang Pan, Min He, Qingyuan Li, Weihong Kuang, Song Wang, Qiyong Gong

**Affiliations:** 1 Huaxi MR Research Center (HMRRC), Department of Radiology, West China Hospital of Sichuan University, Chengdu, 610041 China; 2 Research Unit of Psychoradiology, Chinese Academy of Medical Sciences, Chengdu, 610041 China; 3 Functional & Molecular Imaging Key Laboratory of Sichuan Province, West China Hospital of Sichuan University, Chengdu, 610041 China; 4 School of Public Affairs, Chongqing University, Chongqing, 400044 China; 5 Department of Psychiatry, West China Hospital of Sichuan University, Chengdu, 610041 China; 6 Department of Radiology, West China Xiamen Hospital of Sichuan University, Xiamen, 361000 China

**Keywords:** Psychiatric disorders, Diagnostic markers, Neuroscience

## Abstract

Although functional and structural abnormalities in brain regions involved in the neurobiology of fear and anxiety have been observed in patients with social anxiety disorder (SAD), the findings have been heterogeneous due to small sample sizes, demographic confounders, and methodological differences. Besides, multimodal neuroimaging studies on structural-functional deficits and couplings are rather scarce. Herein, we aimed to explore functional network anomalies in brain regions with structural deficits and the effects of structure-function couplings on the SAD diagnosis. High-resolution structural magnetic resonance imaging (MRI) and resting-state functional MRI images were obtained from 49 non-comorbid patients with SAD and 53 demography-matched healthy controls. Whole-brain voxel-based morphometry analysis was conducted to investigate structural alterations, which were subsequently used as seeds for the resting-state functional connectivity analysis. In addition, correlation and mediation analyses were performed to probe the potential roles of structural-functional deficits in SAD diagnosis. SAD patients had significant gray matter volume reductions in the bilateral putamen, right thalamus, and left parahippocampus. Besides, patients with SAD demonstrated widespread resting-state dysconnectivity in cortico-striato-thalamo-cerebellar circuitry. Moreover, dysconnectivity of the putamen with the cerebellum and the right thalamus with the middle temporal gyrus/supplementary motor area partially mediated the effects of putamen/thalamus atrophy on the SAD diagnosis. Our findings provide preliminary evidence for the involvement of structural and functional deficits in cortico-striato-thalamo-cerebellar circuitry in SAD, and may contribute to clarifying the underlying mechanisms of structure-function couplings for SAD. Therefore, they could offer insights into the neurobiological substrates of SAD.

## Introduction

Social anxiety disorder (SAD) is a prevalent and disabling psychiatric disorder characterized by notable and persistent fear or anxiety in social situations [1]. People with SAD are intensely afraid of possible scrutiny and negative evaluation by others and gradually avoid participating in social activities, resulting in emotional, cognitive, and behavioral disabilities, as well as severe social function impairments [2]. Approximately 7.1–12.1% of people are estimated to suffer from SAD in their lifetime [1], and approximately 90% of SAD patients have at least one comorbid disorder [3]. Given the severe functional impairments of SAD, it is of great importance to understand its neuropathology and identify potential neural biomarkers, which may be crucial for achieving early diagnosis and timely intervention.

Over the last two decades, a large body of neuroimaging (particularly magnetic resonance imaging (MRI)) research has begun to explore the structural and functional abnormalities in SAD, but the results are heterogeneous and in need of validation and replication [4, 5]. On the one hand, evidence from structural MRI (sMRI) studies regarding SAD has indicated a widespread pattern of gray matter (GM) differences in a majority of cortical and subcortical regions, as well as the cerebellum [6–22]. Notably, those findings showed much heterogeneity, to which many confounding issues, such as demographic and methodological discrepancies, may contribute. Specifically, high comorbidity in SAD may significantly complicate the clinical course and diagnosis and make it challenging to study the pure and specific neuropathology of SAD. Nevertheless, different proportions of SAD patients comorbid with different psychiatric disorders have been included, but the effects of comorbidity have not been well handled in previous studies. Besides, previous sMRI studies have typically involved a small sample size of participants with SAD, with the majority of studies including fewer than 30 patients, and few studies justified the sample size or conducted a power calculation. It is well established that studies with small sample sizes are highly susceptible to inflated risks of false positives and negatives [23], and a sample size of 20–30 participants is not sufficient to detect reproducible relationships between the brain and behavior measures regardless of analytic methods [24]. Furthermore, many previous studies on SAD were based on region-of-interest (ROI) analyses, although it has been reported that predefined cerebral ROIs are not isolated due to biological factors, which makes it difficult to correct for multiple comparisons and dramatically increases the risk of type II errors [25]. Hence, to probe the pure neurobiological underpinnings of SAD, it is indispensable and beneficial to explore structural deficits via a whole-brain voxel-based morphometry (VBM) analysis using a sufficient sample size of non-comorbid SAD patients.

On the other hand, functional MRI (fMRI) studies have been performed to determine the functional anomalies in SAD in the context of various emotional, social, and cognitive, as well as other nonspecific tasks [26, 27]. The most consistent findings from these studies were alterations in SAD patients compared to healthy controls (HCs) in the frontolimbic circuitry termed the fear circuitry, which includes hyperactivity in the dorsolateral prefrontal cortex (dlPFC), ventromedial prefrontal cortex (vmPFC), anterior cingulate cortex (ACC), insula, amygdala, and hippocampus/parahippocampus (ParaHIP) [28]. This model posits that dysfunctional top-down modulation is pivotal in the emotional hyperactivity and diminished cognitive processing observed in patients with SAD [29]. Moreover, increasing evidence from some recent studies has revealed other SAD-related functional alterations beyond the conventional fear circuitry [26, 30, 31]. In 2014, a systematic review and meta-analysis conducted by Brühl et al. updated the neurofunctional model of SAD; abnormal fear circuitry in SAD was confirmed, and new findings of hyperactivated medial parietal and occipital regions in response to SAD-related stimuli, as well as hypoconnectivity of parietal, limbic, and executive network regions, were added [26]. Nevertheless, the majority of those functional results were based on task-fMRI using various types of tasks, while it remains to be evaluated whether an analogous pattern of functional anomalies occurs in resting-state brain physiology (i.e., resting-state fMRI (rs-fMRI)), which could offer distinct insights into the intrinsic neurobiology of SAD without the potential confounding effects of task performance [32]. Indeed, the results from the most recent systematic review based on resting-state neuroimaging studies specifically for SAD indicated that the neurobiological substrates of SAD may be, to some degree, different from those classic models (i.e., summarized by Etkin et al.) that primarily originated from task-based studies [33], as this review showed that aberrant (hyper or hypo) connectivity between the amygdala and parietal, temporal, and frontal areas, and abnormal (hypo- or hyper) activity in frontal regions were the most consistently implicated in patients with SAD, as shown by a range of neuroimaging analyses. Additionally, this review suggested that findings from rs-fMRI were confounded by technological and methodological factors and sample characteristics and that further studies with larger samples and consistent analysis methods are warranted.

Furthermore, despite the widespread structural and functional deficits identified in previous studies, multimodal neuroimaging studies on structure-function coupling are relatively scarce in SAD; that is, existing neuroimaging studies on SAD have mainly been performed using a single MRI modality, and few multimodal analyses have been conducted to probe structural and functional deficits and their relationships with the diagnosis of SAD [34–36]. Indeed, some evidence has demonstrated that functional alterations in brain regions are accompanied by structural changes in the corresponding areas [37, 38], and that functional connectivity (FC) and networks could be predicted by structural substrates [39]. A previous study reported that rs-fMRI parameters were variable and may exist where there were no direct structural connections, but their persistence, strength, and spatial statistics were confined by the underlying anatomical structure of the human brain [40]. In other words, structural connections shape and place constraints on FC across the brain network at various spatial scales [41]. As a result, normal structure-function coupling is vital for the brain, while the disrupted coupling of structure and function can be found in many neurological and psychiatric disorders [42–44]. Based on this evidence, a “brain structure-function behavioral coupling” psychoradiological hypothesis indicates that structural alterations in the brain may give rise to clinical syndromes via an impact on disrupted FC [45, 46]. Therefore, a study combining sMRI and fMRI is likely to offer more information on the underlying relationships among brain structure, function, and SAD diagnosis.

Taking these issues into consideration, the present study aimed to determine resting-state FC (rs-FC) alterations in brain areas with structural deficits in non-comorbid adult patients with SAD and to explore potential mechanisms of structural-functional couplings for SAD. To achieve these goals, sMRI and rs-fMRI analyses were conducted in the current study. For structural analysis, an optimized and standardized VBM-Diffeomorphic Anatomical Registration Through Exponentiated Lie algebra (DARTEL) procedure was conducted to measure the GM volume (GMV) [47], a well-validated and widely used index to investigate the neurostructural signatures of GM, which may represent the numbers and sizes of glial cells, unmyelinated neurons, and the volume of the synapses [48]. Turning now to rs-fMRI analysis, a seed-based rs-FC metric that reflects temporal correlations or couplings (i.e., synchronous and coherent fluctuation) of neuronal activity patterns between specific regions (i.e., seeds) with other spatially segregated areas was investigated [49, 50]. As we intended to explore structural-functional couplings, seed-based rs-FC analysis is suitable for the current study, that is, regions with structural deficits could be a priori seeds. In view of existing findings, we hypothesized that a cohort of patients with SAD, compared to a group of HCs, may show altered GMV mainly in subcortical nuclei such as the striatum [16, 21] and abnormalities in large-scale cortical-subcortical circuitry. Considering the sparse and mixed results (i.e., increased vs. decrease) on SAD-related GM change [26, 33], we do not intend to hypothesize the alteration direction. In addition, it could be speculated that the intrinsic functional networks at rest may mediate the correlations between structural deficits and SAD diagnosis.

## Subjects and methods

### Participants and procedures

All procedures in the present study adhered to the ethical standards of the Declaration of Helsinki and the ethical principles in the Belmont Report. This study was approved by the Medical Research Ethics Committee of West China Hospital at Sichuan University. Prior to the experiment, written informed consent was obtained from all participants after they were given a full explanation of the procedures.

This study included 49 right-handed adult SAD patients without any comorbid psychiatric disorders. Patients were recruited from the Mental Health Center of the West China Hospital at Sichuan University. The diagnosis of SAD was confirmed by the consensus of two experienced clinical psychiatrists in accordance with the criteria of Diagnostic and Statistical Manual of Mental Disorders, Fourth Edition (DSM-IV) through the Structured Clinical Interview for DSM Disorders (SCID) [51]. As the power analysis using G Power software [52] indicated that we needed a sample of at least 102 participants to detect a medium-sized effect (Cohen’s d = 0.5, α = 0.05, 1-β = 0.8) to conduct a two-sample *t*-test, 53 HCs were recruited from the local community through advertisements and were matched to the patients in terms of sex, age, and handedness for comparison analysis, and the SCID-Non-Patient Version was conducted to confirm the lifetime absence of psychiatric and neurological illness. The exclusion criteria for all participants were as follows: 1) comorbidity with other axis I psychiatric disorders; 2) axis II antisocial or borderline personality disorders (verified by the SCID); 3) a history of substance dependence or abuse; 4) learning or developmental disorders; 5) a history of head injury; 6) the presence of major neurological or physical diseases; 7) family history of mental disorders; and 8) current pregnancy or claustrophobia and other contraindications to MRI examination. Individuals were also excluded if they were aged under 18 or over 60 years to minimize age-related effects.

Illness onset was determined as the period between the first reported/observed alterations in psychological/behavior state to the development of disease when the patients participated in the study [53], with the information provided by patients, their family members, and medical records. To evaluate and compare the levels of social anxiety between SAD patients and HCs, the self-administered Liebowitz Social Anxiety Scale (LSAS) [54] was administered to assess all participants. As the most commonly used clinical scale in SAD studies, the 24-item LSAS provides scores for both fear factor (LSASF) and social avoidance factor (LSASA), and the total score (LSAST) is their sum, which has shown good validity and reliability in Chinese populations [55].

### Image acquisition and preprocessing

#### Image acquisition

Whole-brain structural and functional MRI images were acquired on a 3.0 T MR scanner (Siemens Trio, Erlangen, Germany) with a 12-channel head coil. During the scans, the subjects were instructed to keep their eyes closed, relax but not to sleep, and lie as still as possible. Earplugs were used to reduce scanner noise, and foam pads were used to restrict head motion as much as possible. High-resolution three-dimensional T1-weighted images were acquired using a spoiled gradient-recalled sequence: repetition time (TR)/echo time (TE) = 1900 ms/2.26 ms, flip angle = 9°, 176 sagittal slices, slice thickness = 1 mm, field of view (FOV) = 240 × 240 mm^2^, data matrix = 256 × 256, voxel size = 1 × 1×1 mm^3^, and in-plane resolution = 0.94 × 0.94 mm^2^. The rs-fMRI data were obtained with the gradient echo-planar imaging sequence: TR/TE = 2000 ms/30 ms; flip angle = 90°; acquisition matrix = 64 × 64; FOV = 240 × 240 mm^2^; thickness = 5.0 mm, without gap; voxel size = 3.75 × 3.75 × 5 mm^3^; and 205 volumes. Each scan was inspected by an experienced neuroradiologist to rule out visible movement artefacts and gross structural abnormalities before image preprocessing.

#### Image preprocessing

Preprocessing of structural images was performed using Statistical Parametric Mapping software (SPM12; Welcome Department of Cognitive Neurology, London, UK; http://www.fil.ion.ucl.ac.uk/spm/) [56]. First, all MRI images were manually reoriented on the anterior-posterior commissure line for better registration. Second, the high-resolution T1-weighted images were segmented into GM, white matter (WM), and cerebrospinal fluid (CSF) via the new segmentation tool in SPM12. Third, the GM data were aligned, resampled to 2 × 2 × 2 mm^3^, normalized to Montreal Neurological Institute (MNI) space, modulated for the preservation of GMV, and smoothed with an 8-mm full-width at half-maximum (FWHM) Gaussian kernel using DARTEL in SPM12 [57].

The rs-fMRI data were preprocessed using the Data Processing Assistant for Resting-State fMRI (DPARSF 4.3, http://rfmri.org/DPARSF), which is based on SPM (http://www.fil.ion.ucl.ac.uk/spm/) and the toolbox for Data Processing & Analysis of Brain Imaging (DPABI 4.3, http://rfmri.org/DPABI) [58] and includes the following steps: 1) removal of the first 10 volumes and slice timing correction; 2) realignment and correction for head motion (three SAD patients and one HC with excessive head motion above 2.5 mm or 2.5° in any direction were excluded), in which the framewise displacement (FD) was calculated for subsequent analysis; 3) spatial normalization to MNI space including the new segmentation and DARTEL; 4) regressing out linear trends, Friston’s 24 head motion parameters [59], WM signal, CSF signal, and global signal; 5) resampling into 3 × 3 × 3 mm^3^ and spatial smoothing with a 6-mm FWHM Gaussian kernel; 6) temporal bandpass filtering (0.01–0.08 Hz); and 7) calculating rs-FC based on the clusters showing significant group differences in the VBM analysis as the seed areas. The time courses were extracted from the seed areas, and the correlation coefficients between those time courses and all remaining brain voxels were computed. Finally, the correlation maps were z-normalized using Fisher’s r-to-z transformation to improve the normality of the partial correlation coefficients.

### Statistical analysis

#### Demographic and clinical data analyses

The differences in the demographic and clinical data between two groups were conducted through a chi-square test for discrete variables (i.e., sex) and two-sample t-tests for continuous variables (i.e., age, LSAST, LSASA, LSASF, and mean FD) using IBM SPSS Statistics 22.0.

#### VBM and FC analyses

Whole-brain voxel-wise comparisons of GMV between groups were performed using two-sample *t*-tests, with age, sex, and total intracranial volume (TIV) as covariates of no interest in SPM12. Group differences in rs-FC were conducted in DPABI software, in which two-sample *t*-tests were used to compare rs-FC between SAD patients and HCs with age, sex, and mean FD as covariates of no interest. The Gaussian random field (GRF) theory [60, 61] was performed to control for multiple comparisons with a significance threshold of a voxel-wise value of *P* < 0.001 and cluster probability of *P* < 0.05 [62]. In addition, we applied the false discovery rate (FDR) to correct for four seeds in the FC analyses.

#### Correlation analyses

To identify the associations between the structural/functional changes and clinical characteristics, the average GMV and rs-FC values in the significant clusters with between-group differences were extracted respectively, and then we performed a partial correlation analysis between the aforementioned mean values and clinical features (i.e., LSAST, LSASA, LSASF, and disease duration) using sex, age, and TIV/mean FD as covariates in the SAD group via IBM SPSS Statistics 22.0.

#### Mediation analysis

To explore the potential mediating roles of functional deficits in the relationship between the structural abnormalities and SAD diagnosis, a mediation analysis was performed with the SPSS macro PROCESS that included the bootstrapping approach [63, 64]. To this end, GMV of the brain regions with significant between-group differences was considered the independent variable (X); corresponding FC of identified regions was the mediator variable (M); SAD diagnosis served as the dependent variable (Y); and age, sex, TIV, and mean FD were regarded as covariates. Then, mediation analysis was conducted to investigate the direct (i.e., path c’ representing the relationship between X and Y after controlling for M) and indirect relationships between structural deficits and SAD diagnosis. The indirect effect represented the product of path a (i.e., the relationship between X and M) and path b (i.e., the relationship between M and Y after adjusting for X). The estimation of the indirect effect was considered significant if zero was not included in the bootstrapped 95% confidence intervals (CIs) (number of samplings= 5000).

## Results

### Demographic and clinical characteristics

One hundred and two participants (49 SAD vs. 53 HC) were included in the VBM analysis, while 98 subjects (46 SAD vs. 52 HC) were included in the rs-FC-related analysis because of the removal of 4 participants (3 SAD vs. 1 HC) due to head motion. No significant group differences appeared in terms of sex composition and age; the patients with SAD had significantly higher LSAS scores than the HCs (Table 1). Besides, there were no significant differences in mean FD between the two groups [*t* (98) = 0.519, *P* = 0.605].

**Table 1:** Demographics and clinical characteristics of participants.

Characteristics	SAD (*N* = 49)	HCs (*N* = 53)	*P* value
**Sex (Male/Female)**	30/19	31/22	0.778*
**Age (years)**	24.6 ± 5.3 (18–38)	23.4 ± 3.3 (18–35)	0.194**
**Illness duration (years)**	7.2 ± 4.1 (1–20)	–	–
**LSAST**	64.5 ± 23.8 (23–115)	18.5 ± 8.4 (1–30)	<0.001**
**LSASF**	31.9 ± 11.7 (13–57)	10.2 ± 5.4 (1–22)	<0.001**
**LSASA**	32.2 ± 13.0 (9–62)	8.3 ± 6.0 (0–27)	<0.001**

*FD* framewise displacement, *HCs* healthy controls, *LSAST, LSASF, and LSASA* total score and fear and avoidance factor scores on the Liebowitz Social Anxiety Scale (LSAS), *SAD* social anxiety disorder.

Data are presented as the means ± standard deviations (minimum−maximum).

*P value obtained using a chi-square test.

**P value obtained using a two-sample *t*-test.

### Group differences in GMV

**Fig. 1:**
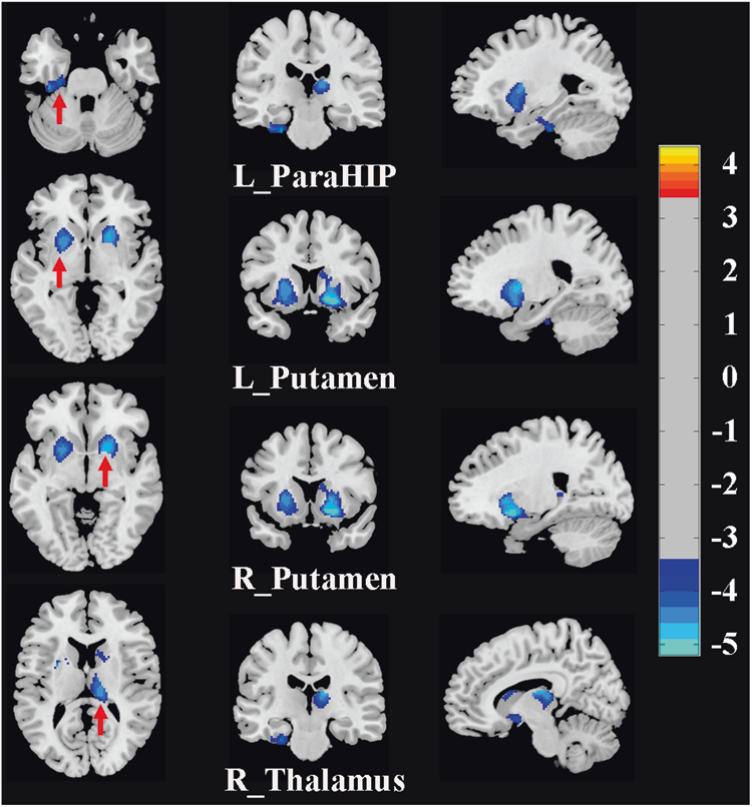
Brain regions with significant differences in GMV between SAD patients and HCs (corrected with Gaussian random field theory with a significance threshold of a voxel-wise value of *P* < 0.001 and cluster probability of *P* < 0.05). Warmer colors (positive values) represent increased GMV, while cooler colors (negative values) represent decreased GMV in SAD patients compared to HCs. Abbreviations: GMV, gray matter volume; HCs, healthy controls; L, left; ParaHIP, parahippocampus; R, right; SAD, social anxiety disorder.

The whole-brain voxel-wise analysis demonstrated that the SAD patients, compared to the HCs, had significantly decreased GMV in the right thalamus, bilateral putamen, and left ParaHIP. No areas showed larger GMV in the SAD group (Fig. 1 and Table 2).

**Table 2:** Brain regions with significant differences in GMV and rs-FC between SAD patients and HCs.

Significant clusters	Peak MNI coordinate of significant clusters			Cluster size (voxels)	Peak *T* value
	X	Y	Z		
**Between-group differences in GMV**					
**SAD** < **HCs**					
Left ParaHIP	−26	−24	−32	153	−4.667
Left putamen	−12	−6	18	820	−4.722
Right putamen	20	10	−10	828	−5.197
Right thalamus	12	−20	16	299	−4.975
**SAD** > **HCs**					
none					
**Between-group differences in rs-FC**					
**Seed1: Left putamen**					
**SAD** > **HCs**					
Left MTG/STG	−63	−42	−3	173	4.810
**SAD** < **HCs**					
Left cerebellum	−39	−57	−24	59	−4.999
**Seed2: Right putamen**					
**SAD** > **HCs**					
Left STG/MTG	−57	-60	27	50	4.593
**SAD** < **HCs**					
Right cerebellum	18	−90	-33	159	−4.465
**Seed3: Right thalamus**					
**SAD** > **HCs**					
Left MTG	−48	−63	21	83	4.433
Right MTG/STG	57	−60	21	113	5.027
Right ITG/fusiform gyrus	48	−51	−12	60	4.291
**SAD** < **HCs**					
Limbic Lobe/Left ACC	−12	24	30	190	−5.227
Left SMA/SFG	−3	−6	72	62	−4.997
Left thalamus	−9	−12	18	143	−5.195
cerebellum	0	−75	−15	126	-4.392

*ACC* anterior cingulate cortex, *GMV* gray matter volume, *HCs* healthy controls, *ITG* inferior temporal gyrus, *MNI* Montreal Neurological Institute, *MTG* middle temporal gyrus, *ParaHIP* parahippocampus, *rs-FC* resting-state functional connectivity, *SAD* social anxiety disorder, *SFG* superior frontal gyrus, *SMA* supplementary motor area, *STG* superior temporal gyrus.

All clusters survived correction for multiple comparisons using Gaussian random field theory with a significance threshold of a voxel-wise value of *P* < 0.001 and cluster probability of *P* < 0.05.

### Group differences in FC

**Fig. 2:**
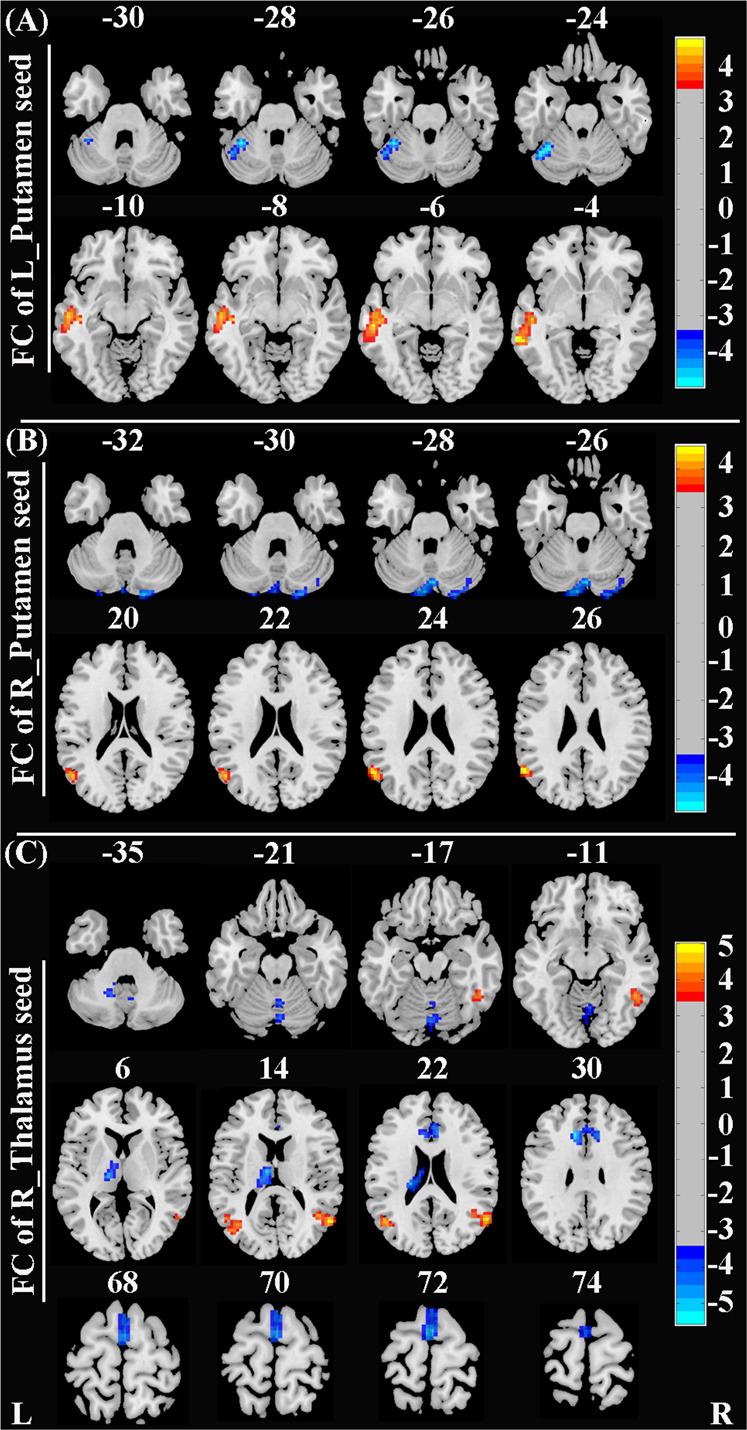
Brain regions with significant differences in rs-FC between SAD patients and HCs (corrected with Gaussian random field theory with a significance threshold of a voxel-wise value of *P* < 0.001 and cluster probability of *P* < 0.05). Warmer colors (positive values) represent increased rs-FC, while cooler colors (negative values) represent decreased rs-FC in SAD patients compared to HCs. Abbreviations: HCs, healthy controls; L, left; R, right; rs-FC, resting-state functional connectivity; SAD, social anxiety disorder.

Compared to the HCs, patients with SAD had increased rs-FC between the left putamen and left middle temporal gyrus (MTG)/superior temporal gyrus (STG), and decreased rs-FC between the left putamen and left cerebellum; increased rs-FC between the right putamen and left STG, and decreased rs-FC between the right putamen and right cerebellum; increased rs-FC between the right thalamus and bilateral MTG/STG and right inferior temporal gyrus (ITG), and decreased rs-FC between the right thalamus and limbic lobe/ACC, supplementary motor area (SMA)/superior frontal gyrus (SFG), bilateral cerebellum, and left thalamus (Fig. 2 and Table 2). When the seed area was located in the left ParaHIP, there were no regions with significantly different rs-FC.

### Correlations between structural/functional deficits and clinical characteristics

After controlling for the confounders of sex, age, TIV, and mean FD, the partial correlation analysis showed that decreased GMV in the bilateral putamen was significantly inversely related to SAD duration (left: *r* = −0.34, *P* = 0.021; right: *r* = −0.407, *P* = 0.005), while decreased rs-FC between the right thalamus and limbic lobe/ACC (*r* = 0.355, *P* = 0.020) and decreased rs-FC between the right thalamus and cerebellum (*r* = 0.321, *P* = 0.036) were positively correlated with SAD duration (*see* Supplementary Materials). There was no significant association between the structural/functional alterations and LSAS scores.

### Mediation analyses

**Fig. 3:**
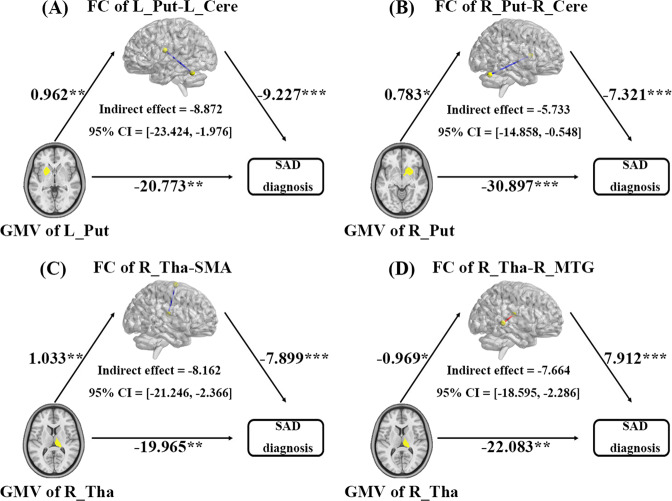
Mediating role of rs-FC deficits on the effects of GMV abnormalities on SAD diagnosis. Unstandardized regression coefficients are displayed (*P < 0.05, **P < 0.01, ***P < 0.001). Age, sex, total intracranial volume, and mean framewise displacement were controlled for in the model. Abbreviations: CI, confidence interval; GMV, gray matter volume; HCs, healthy controls; L_Cere, left cerebellum; L_Put, left putamen; R_Cere, right cerebellum; R_MTG, right middle temporal gyrus; R_Put, right putamen; rs-FC, resting-state functional connectivity; R-Tha, right thalamus; SAD, social anxiety disorder; SMA, supplementary motor area.

In the mediation analysis, we found a significant mediating effect of decreased rs-FC between the left putamen and left cerebellum on the association between decreased GMV in the left putamen and SAD diagnosis (indirect effect = −8.872, 95% CI = [−23.424, −1.976], *P* < 0.05]; Fig. 3A); meanwhile, decreased rs-FC between the right putamen and right cerebellum was observed to play a mediating role in the relationship between decreased GMV in the right putamen and SAD diagnosis (indirect effect = −5.733, 95% CI = [−14.858, −0.548], *P* < 0.05; Fig. 3B). In addition, dysconnectivity of the right thalamus with the SMA (indirect effect = −8.162, 95% CI = [−21.246, −2.366], *P* < 0.05; Fig. 3C) or the right MTG (indirect effect = −7.664, 95% CI = [−18.595, −2.286], *P* < 0.05; Fig. 3D) played a significant mediating role in the relationship between decreased GMV in the right thalamus and SAD diagnosis.

## Discussion

In the present study, we demonstrated that SAD patients had structural and functional deficits in the cortico-striato-thalamo-cerebellar circuitry and uncovered significant mediating effects of functional anomalies on the links between structural deficits and SAD diagnosis. To our knowledge, the current study was the first to combine VBM and rs-FC to reveal structural and functional deficits and couplings in relation to SAD, which may be integral to the neuropathology of SAD and to some degree contribute to the future early diagnosis and targeted therapy in SAD.

### Cortico-striato-cerebellar circuitry in SAD

First, the current study pointed to the dysfunctional cortico-striato-cerebellar circuitry in SAD. The VBM analysis revealed decreased GMV in the bilateral putamen in SAD patients compared to HCs, while a recent multicentre mega-analysis showed that patients with SAD had larger GMV in the putamen [16]. Without regard to the alteration directions, those findings at least indicated the involvement of putamen in SAD [65]. Robust evidence has accumulated that the putamen (i.e., a part of the dorsal striatum) is involved in social learning, motor, and cognitive control, reward processing, and cognitive and emotional regulation [66, 67]. It has been documented that SAD patients lack a processing preponderance in the putamen for social rewards compared to social punishments [68], so structural alterations in the putamen may be responsible for its involvement in the imbalance of the neural approach-avoidance motivation system underlying SAD. Meanwhile, increased FC between the putamen and the MTG/STG and decreased FC between the putamen and cerebellum posterior lobe were also presented in the current analysis. The STG/MTG is a crucial component of the perceptual system involved in facial emotion processing, social threat evaluation [69], analysis of the dispositions and intentions of others’ actions [70, 71], visual perception and mental imagery [72], and integration of interoceptive information with information about the current environmental situation [73], all of which may be related to SAD characteristics such as an excessive focus on others’ intentions and facial expressions, excessive fear for negative evaluation and scrutiny by others [74], and increased saliency of the social situations when SAD patients envision themselves in hypothetical scenes [75]. Interestingly, a recent study also reported increased intrinsic rs-FC in the left MTG in families genetically enriched for SAD, indicating the crucial roles of the MTG as a network hub in the socially anxious brain [76]. Combined with the findings that striatal dysfunction was closely related to the information processing biases in SAD [77], increased FC between the putamen and the MTG/STG may reflect enhanced input of undue focus and speculation on social and individual stimuli into the striatum and subsequent cognitive and emotional dysregulation.

Another interesting finding was decreased FC between the putamen and cerebellum posterior lobe in SAD patients. Not surprisingly, cerebellar structural and functional anomalies have been implicated in the emotional dysregulation associated with various psychiatric disorders [78–80], especially anxiety-related symptoms (e.g., hyperarousal) and psychosis [11, 81–83], and abnormal resting-state cerebellar activity and cerebellum-based FC were observed in patients with SAD [84, 85]. Indeed, the cerebellum has traditionally been considered a region exclusively involved in motor control and coordination [86], but recently, its involvement in nonmotor domains, such as emotion regulation [87], cognitive processing (i.e., visual-spatial, executive, and working memory) [88], and reward-related learning [89], has drawn much attention. Convergent evidence indicates that dysconnectivity between the putamen and cerebellar lobules is implicated in various cognitive functions and is interconnected with the default mode and frontoparietal networks [90, 91]. Therefore, we hypothesized that diminished putamen-cerebellar connectivity may lead to dysfunctional cognitive and emotional regulation in patients with SAD.

### Cortico-thalamo-cerebellar circuitry in SAD

Current research has also pointed to structural/functional deficits in cortico-thalamo-cerebellar circuitry. In agreement with a recent meta-analysis of VBM studies [92, 93], we found that patients with SAD had lower volumes in the right thalamus and left ParaHIP, crucial components of limbic structures whose structural and functional abnormalities were found in SAD studies [8, 94–96]. It has been documented that the thalamus and ParaHIP are implicated in emotion regulation, emotional salience, and cognitive/executive networks [97]; dysfunction in the thalamus, as a part of the arousal system, may be related to hypersensitivity and hypervigilance to social stimuli and emotional dysregulation [98], while ParaHIP deficits in SAD may reflect disrupted contextual fear conditioning and failure to assign accurate saliency value to stimuli [99]. Hence, it is speculated that emotional and cognitive/executive dysfunction may be linked to progressive atrophy of the thalamus and ParaHIP in SAD.

From a network perspective, the cerebellum is closely connected with both motor and nonmotor (cognitive and affective) cortical regions via feedback projections of the cerebellum [88], both motor and nonmotor thalamic nuclei receive outputs from the cerebellum [90], and dysfunction in the cortical-thalamic-cerebellar circuit could damage the efficiency of receiving input and producing output [100]. Furtherly, evidence indicated that cortical-(para)limbic imbalance is one of the core pathophysiologies of SAD in which the PFC/ACC fails to adequately mediate the limbic regions, which then demonstrate dysfunctional activity [26, 101–103], while current study also found the decreased connectivity between right thalamus and PFC/limbic lobe/ACC. In this sense, this evidence aligns well with current findings of decreased connectivity within the cortical-thalamic-cerebellar circuitry. In addition, the SAD patients showed decreased connectivity within the SMA-thalamic-cerebellar circuitry in this study. A study in healthy participants reported that the early binding of gaze, gestures, and emotions is achieved in the motor system (e.g., SMA and premotor cortex), which may prompt the preparation of an adaptive response to another person’s immediate intention [104], while gaze avoidance towards emotional stimuli is one of the important characteristics in SAD patients [105].

Taken together, evidence is accumulating for the interconnection of the cortex, basal ganglia, and thalamus in large-scale loops (i.e., cortico-striato-thalamo-cortical circuitry; CSTCort circuitry) and for their involvement in vital cerebral function [106, 107], which is considered a prevailing model regarding the neural and pathophysiological underpinnings of obsessive-compulsive disorder (another important anxiety-related psychosis in DSM-IV) [108, 109]. The current study not only confirmed the involvement of CSTCort circuitry in SAD but also highlighted the crucial roles of the cerebellum in SAD, thus indicating that aberrant cortico-striato-thalamo-cerebellar (CSTCere) circuitry may contribute to the psychopathological and pathophysiological basis of SAD; that is, dysfunctional CSTCere circuitry may contribute to the undue appraisal of external stimuli such as facial emotion and potential social threat, defective cognitive, emotional, and social motor processing, and consequent excessive fear and avoidance of social interactions, which contribute to the occurrence and development of SAD.

### Couplings of structural and functional deficits in the prediction of SAD diagnosis

Furthermore, the subsequent analysis identified that functional deficits may partially mediate the influence of structural abnormalities on SAD diagnosis. Previous studies have reported widespread structural and functional abnormalities [26, 27, 31, 33], and a considerable number of regions with structural anomalies were compatible with functional deficits, some of which have shown great accuracy for clarifying SAD patients and HCs [31, 110], suggesting that structural and functional deficits may be the pathophysiological bases and could serve as potential biomarkers for SAD. Furtherly, as a relatively more stable variable, structural features (e.g., GMV) are generally deemed the basis of functional parameters (e.g., rs-FC), and functional characteristics may be outward manifestations resulting from structural changes [40]. As previous studies demonstrated, between-group differences in functional activation in certain regions showed much overlap with the structural alterations [35], and a wide range of areas could conform to the principle of “the greater the GM concentration, the greater the task-related activation change from baseline” [38]. Besides, spatial statistics, strength, and persistence of rs-FC, are determined by the large-scale cerebral structural backbone, indicating a close interrelation of brain structure and function [40, 111]. In agreement with this, our mediation analysis confirmed that dysconnectivity based on the bilateral putamen and right thalamus seeds could partially mediate the relationships between atrophy of subcortical nuclei and SAD diagnosis. In other words, we speculated that atrophy of the bilateral putamen and right thalamus may cause aberrant functional synchronization and then give rise to defective function in some vital areas, eventually leading to the occurrence of SAD.

As a result, current findings may provide novel insights into the pathophysiological and neurobiological substrates underlying SAD; that is, the corresponding functional deficits may be a potential intrinsic mechanism linking GMV alterations to SAD occurrence. In this sense, our results may support the view that those identified structural and functional alterations were more closely related to the category of disorder than the psychopathological dimension [20]. To some degree, this speculation may be further confirmed by our exploratory correlation analysis, as we failed to detect significant correlations between those neuroanatomical differences and symptom severity. Nevertheless, it should be mentioned that some previous studies indeed observed relationships between neuroanatomical alterations and SAD symptom severity [6, 9, 10, 16], indicating that further studies need to be conducted to investigate this exact relationship. Instead, we observed significantly negative correlations between decreased GMV in the bilateral putamen and SAD duration and positive correlations between dysfunctional rs-FC of the right thalamus with limbic lobe/ACC or cerebellum and SAD duration. Our results indicated that as the disease course was extended, SAD patients experienced disrupted bottom-up processes and top-down control in response to external stimuli evoking social anxiety, thus suffering from more severe damage to the subcortical nuclei and cortical-subcortical functional circuitry that appeared as resultant subcortical atrophy and large-scale circuit dysconnectivity.

### Comparison between our results and previous findings and potential implications

Along with the rapid growth of neuroimaging studies of SAD, increasing attention has been given to the validation and replication of findings that are vital for clinical transformation. Indeed, the neuroimaging results on SAD have been of limited consistency. Previous sMRI and rs-fMRI studies, which have been summarized in [21, 33], have reported a widespread but variable pattern of brain regions with GM structural and functional alterations, while the current study detected GMV alterations mainly in subcortical regions and functional deficits in large-scale CSTCere circuitry. The differences from our results may be attributed to several factors. From a methodological perspective, first, we used automated software SPM with optimized and standardized DARTEL procedure, a validated VBM method with good test-retest reliability, for preprocessing and statistical analysis, while manual segmentation or less accurate methods were performed in some previous studies [19, 112, 113]. Second, we used whole-brain voxel-wise analysis, different from the ROI analysis adopted in some other studies, which may significantly increase the risk of Type II error [25], and could be more sensitive to detect alterations especially for small structures such as the amygdala due to multiple comparison corrections [114]. Third, GMV is a complicated parameter that is different from other surface-based indices, such as cortical thickness, surface area, and cortical folding (gyrification index) [115]. Another reasonable interpretation for the discrepancies is that no changed GMV may be the result of cortical thinning with concurrent surface expansion or vice versa [115], as our recent study demonstrated dissociations in cortical thickness and surface area (i.e., decreased cortical thickness and increased cortical surface area) in SAD patients [22, 116]. Fourth, differences in parameter settings on preprocessing and statistical analysis may also contribute to the heterogeneity. For instance, the definition of seeds (e.g., choosing a prior mask of brain areas with structural/functional abnormalities vs. a combination of coordinates and radius) may also cause different results. From the perspective of samples, demographic variations, such as disease severity, illness durations, and comorbidities can confound results [117, 118]. Another nonnegligible reason for inconsistent results is the sample size, which is elaborated earlier (Introduction section). In summary, many aspects of the methodological, medical, and sociodemographic domains are associated with (or perhaps cause) neurostructural/functional alterations in SAD.

Neuroscience plays a crucial role in a translational approach to inform the improvement and development of diagnostic and therapeutic strategies. In the future, along with the identification and confirmation of the close (even causal) relation between the GM structural/functional alterations and occurrence/progression of SAD, those identified regions could serve neural biomarkers for early diagnosis of SAD, as well as reliable and noninvasive tools for disorder prognosis and treatment efficacy assessment [31, 116, 119]. Furthermore, regions with GM abnormalities are potential therapeutic targets. These findings may not only contribute to the selection, optimal use, refinement, or development of targeted drugs, but also direct modulation of SAD-specific areas via nonpharmacological neurobiological interventions such as deep brain stimulation [120], repetitive transcranial magnetic stimulation [121, 122], and real-time functional MRI neurofeedback [123], which may be promising choices in the future. For instance, based on accumulated evidence from neuroimaging studies highlighting the crucial roles of the lateral-medial PFC in SAD, a recent randomized, double-blind, and parallel-group study demonstrated that transcranial direct current stimulation over the dlPFC and mPFC can significantly alleviate SAD core symptoms (i.e., fear and avoidance), reduce attention bias to threat-related stimuli, and improve therapy-related variables (i.e., emotion regulation, depressive state, worries, and quality of life) [124]. In summary, neuroimaging studies could offer further insights into the neurobiological mechanisms of SAD, which is of vital importance for guiding effective diagnosis and therapy to improve the quality of life of SAD patients as much as possible [31]; this is also the aim of psychoradiology [125,126]. Notwithstanding many important efforts, we should also recognize that there is still a long way from bench to bed. It remains to be seen to what extent those neuroimaging results based on the symptom-based diagnostic categories for psychiatric disorders could reflect the specific pathophysiological mechanisms and their relations to clinical symptoms. Furthermore, future research can benefit much from investigating social anxiety dimension-based measures in combination with longitudinal studies of interventional effects.

### Limitations

When interpreting the current findings, some limitations deserve mentioning. First, it needs to be clarified whether our results could have been influenced by examination-related anxiety during the MRI scans. To do this, we would need to assess the psychological reactions and psychophysiological responses of participants before/during/after the MRI examination [127]. Second, as a cross-sectional design cannot explicitly elucidate the causal relationships between structural/functional abnormalities and disease state, future longitudinal and developmental studies involving follow-up evaluations and studying people with high innate vulnerability to developing SAD (e.g., based on the genotypes and endophenotypes [128]) will be much more beneficial for providing further insights into the neurobiological and psychopathological underpinning and progression course of SAD. Third, it would have been desirable to measure and, if feasible, to match the patients with HCs on intelligence quotient measures, which showed a positive correlation with brain volume [129]. Nevertheless, as the TIV was included as a covariate in the analysis and there is no definite proof supporting the notion that SAD patients are subject to intellectual impairment, the effects of intelligence on the current findings are quite limited. Fourth, given that SAD typically evolves during late childhood and early adolescence, it remains to be seen whether the current findings based on adult SAD patients could be generalized to adolescent populations. To do this, future researches need to investigate the neuroanatomical alterations in a cohort of child and adolescent patients suffering from SAD. Fifth, although power analysis was adopted to guarantee medium-sized effects (to the best of our knowledge, the current study is thus far the relatively large single-center study investigating whole-brain structural and functional deficits in non-comorbid SAD patients), our sample size is not very large compared to recent studies exploring other psychiatric disorders. This is partly due to strict inclusion criteria of adult SAD patients without any comorbid disorders, with the hope of investigating the pure and specific neurobiology of SAD, which may also to some degree limit the generalizability of our findings. In the future studies, researchers could benefit from exploring the effects of confounded factors on structural/functional deficits, and our results need further replication via a larger sample. Sixth, the cross-sectional design makes it difficult to identify causal associations regarding brain structural/functional alterations and SAD diagnosis. In particular, the exact correlations between cerebral structure and function remain to be investigated. Consequently, we also conducted another mediation design (i.e., *X* = FC, *M* = GMV, *Y* = SAD diagnosis; age, sex, TIV, and mean FD were regarded as covariates), and we observed similar results to the main analysis, i.e., a significant mediating effect of decreased GMV in the bilateral putamen on the association between decreased rs-FC of bilateral putamen with cerebellum and SAD diagnosis respectively (left: indirect effect = −1.748, 95% CI = [−4.768, −0.351], *P* < 0.05]; right: indirect effect = −1.784, 95% CI = [−5.510, −0.155], *P* < 0.05])); a significant mediating role of right thalamus atrophy in the relationship between the dysconnectivity of the right thalamus with the SMA (indirect effect = −1.365, 95% CI = [−3.949, −0.238], *P* < 0.05]) or with the right MTG (indirect effect = 1.490, 95% CI = [0.369, 3.923], *P* < 0.05]) and SAD diagnosis. Therefore, other potential mediating associations may exist among GMV, FC, and SAD diagnosis, although our main analysis attempted to investigate brain structure-function-behavior couplings to disclose the potential neurobiological mechanisms underlying SAD. In summary, considering that current data from the cross-sectional design were not temporally discernable, the mediating analyses conducted in this study were of a statistical or theoretical nature, and the main results may be just a possible mechanism linking GMV, FC, and SAD. To identify the causal relationships of these variables, longitudinal and interventional (e.g., therapeutic trial) designs are needed in future works. Finally, as the results of FC analyses based on the seeds with structural deficits may be partly biased by the seeds selection, future works need to establish structural and functional networks in whole-brain regions to investigate the effects of structural-functional couplings on SAD.

### Conclusions

The current study complemented and extended prior SAD-related neuroimaging studies by identifying the involvement of the CSTCere circuitry in non-comorbid adult patients with SAD and revealing the potential coupling mechanisms of structural and functional deficits in the prediction of SAD diagnosis, which together indicate that the aberrant CSTCere circuitry may contribute to the neurobiological basis of SAD. The current findings might provide insights into understanding the neurobiological substrates of SAD and initial evidence for further identification of candidate neuroanatomical biomarkers, which may advance the early diagnosis, targeted treatment, and therapeutic evaluation for SAD.

## Supplementary information

Figure S1

## Data Availability

The data and code that support the findings of present study are available from the corresponding author through reasonable request. The data and code sharing adopted by the authors comply with the requirements of the funding institute and with institutional ethics approval.
