# Supplementary material for: Structural and functional deficits and couplings in the cortico-striato-thalamo-cerebellar circuitry in social anxiety disorder
Source: Transl Psychiatry. 2022 Jan 21;12:26. doi: 10.1038/s41398-022-01791-7 (PMC8782859; doi:10.1038/s41398-022-01791-7)

**Supplementary Materials**

**Supplementary Figures**

Figure S1. Scatter plots depicting the correlation between illness duration and grey matter volume or functional connectivity in the corresponding areas. The scores on the x-axis represent the standardized residuals of the duration after sex, age, total intracranial volume, or mean framewise displacement were regressed out. The scores on the y-axis represent the standardized residuals of the values of grey matter volume (A-B) or functional connectivity (C-D) in the corresponding areas after sex, age, total intracranial volume, or mean framewise displacement were regressed out. Abbreviations: ACC, anterior cingulate cortex, Cere, cerebellum; FC, functional connectivity; GMV, grey matter volume; R-Tha, right thalamus.


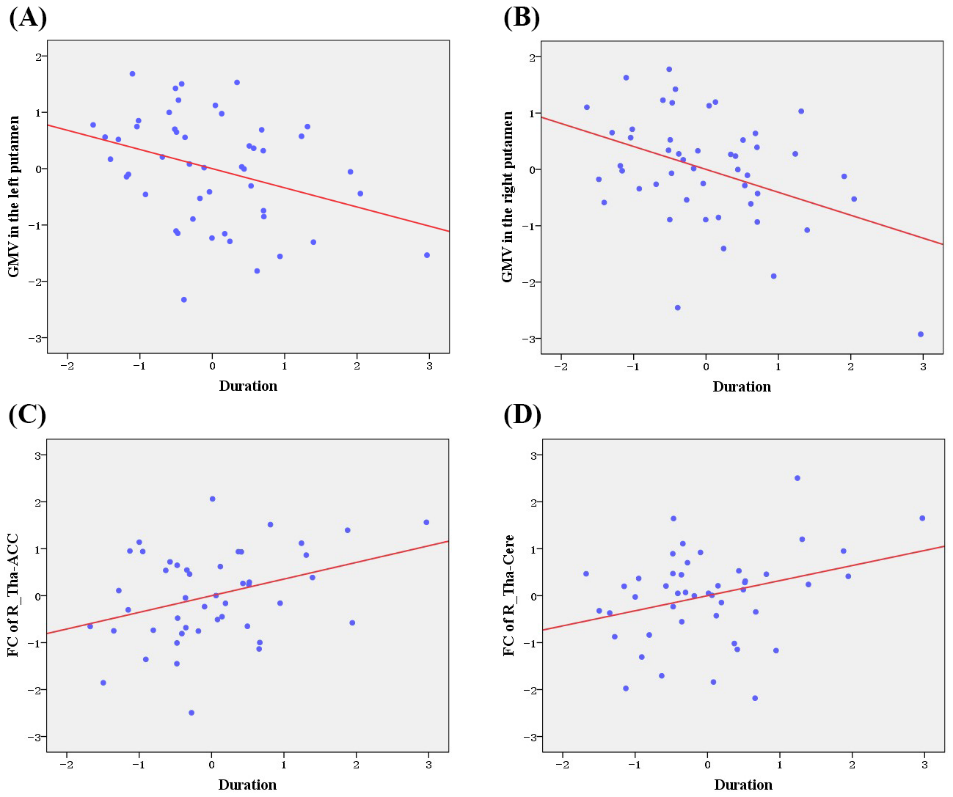

Supplement: Supplementary file 1 — Figure S1 [file 41398_2022_1791_MOESM1_ESM.docx]
